# Translation of ionic liquids to be enteric nanoparticles for facilitating oral absorption of cyclosporine A

**DOI:** 10.1002/btm2.10405

**Published:** 2022-09-07

**Authors:** Kaiheng Liu, Wenjuan Liu, Zirong Dong, Luyu Zhang, Qiuyu Li, Renjie Zhang, Haisheng He, Yi Lu, Wei Wu, Jianping Qi

**Affiliations:** ^1^ Key Laboratory of Smart Drug Delivery of MOE, School of Pharmacy Fudan University Shanghai China

**Keywords:** cyclosporine A, enteric, ionic liquids, mesoporous silica nanoparticles, oral

## Abstract

Ionic liquids (ILs) attract more and more interests in improving drug transport across membrane, including transdermal, nasal, and oral delivery. However, some drawbacks of ILs impede the application in oral drug delivery, such as rapid precipitation of poorly soluble drugs in stomach. This study aimed to employ enteric mesoporous silica nanoparticles (MSNs) to load ILs to overcome the shortcomings faced in oral administration. The choline sorbate ILs (SCILs) were synthesized by choline bicarbonate and sorbic acid and then adsorbed in mesopores of MSNs after dissolving cyclosporin A (CyA). MSNs loading SCILs and CyA were coated by Eudragit® L100 to form enteric nanoparticles. The in vitro release study showed that the CyA and SCILs released only 10% for 2 h in simulated gastric fluids but more than 90% in simulated intestinal fluid. In addition, SCILs and CyA were able to release from MSNs synchronously. After oral administration, enteric MSNs loading SCILs were capable of improving oral absorption of CyA significantly and the oral bioavailability of CyA was similar with that of oral Neoral®. In addition, the oral absorption of enteric MSNs was higher than that of nonenteric MSNs, which showed that enteric coating was necessary to ILs in oral delivery. These findings revealed great potential of translation of ILs to be enteric nanoparticles for facilitating oral absorption of CyA. It is predictable this delivery system is promising to be a platform for delivering poorly water‐soluble drugs and even biologics orally.

## 1 INTRODUCTION

Ionic liquids (ILs) usually refer to ionic compounds with a melting point below 100°C, and those with a melting point at or near room temperature are also called room temperature molten salts.^1^ ILs have been widely used as a new type of green solvents in the fields of chemistry, electrochemistry, and materials due to their advantages of large heat capacity, noncombustibility, low vapor pressure, and good solubility for many compounds.^2^, ^3^ Recently, some biocompatible ILs have also been employed as biological solvents for transdermal and mucosal delivery.^4^, ^5^, ^6^ As we know, oral delivery is the most favorable route for patients. However, a large number of drugs are difficult to exert therapeutic effect by oral administration due to poor water solubility or permeability. Some reports have shown that ILs were capable of improving oral bioavailability of drugs, like insulin and sorafenib.^7^, ^8^ Unfortunately, ILs could dissociate in stomach once encountering enormous gastric fluids.^9^ After dissociation, some poorly water‐soluble drugs will quickly recrystallize and precipitate, while water‐soluble drugs released in gastric fluid completely and could be degraded in harsh gastric environments. As a result, the effects of ILs are not able to exert perfectly, including increasing dissolution and enhancing permeation. Therefore, some studies adopted intrajejunal injection or enteric capsule for administration to animals.^10^ Enteric capsule seems to be the feasible approach for clinical use; however, it could be broken by ILs during storage due to their strong solubilizing capacity and hygroscopicity.^11^, ^12^ Hence, it is urgent to develop a feasible carrier to protect ILs against dissociation in gastrointestinal tract for oral delivery.

Mesoporous silica nanoparticles (MSNs) have gained much attention for oral delivery due to many uniform mesopores, which are able to load drugs, and high dispersity in gastrointestinal tract that is able to inhibit drug recrystallization.^13^ Besides, many reports have confirmed MSNs were capable of penetrating intestinal mucus layer.^14^, ^15^ Thus, we employed MSNs to carry drugs and ILs simultaneously and then coated with enteric materials. Poor water‐soluble drugs are solubilized in ILs and then loaded in mesopores of MSNs by adsorption. The enteric coating protects against precipitation of drug molecules in stomach. After entering small intestine, MSNs could distribute broadly in whole intestinal gut and even penetrate mucus layer. When each nanoparticle took off enteric coat with the increase of pH in small intestine, drugs and ILs would release from mesopores simultaneously, inhibiting precipitation of poor water‐soluble drugs and improving permeation of drugs due to the presence of ILs. Combining with nanotechnology and enteric coating, ILs would fully exert their abilities of enhancing dissolution and permeation of drug molecules in gastrointestinal tract.

Herein, cyclosporine A (CyA) was used as a model drug to verify the feasibility of the idea. CyA is a biopharmaceutics classification system (BCS) IV compound, whose bioavailability is limited by both poor water solubility and lower permeability across intestinal epithelia.^16^ The commonly used product on the market is CyA self‐emulsifying soft capsules (Neoral®), which are obtained by using high‐dose surfactants (polyoxyethylene hydrogenated castor oil 40), corn oil‐mono‐di‐triglycerides and absolute ethanol.^17^ Neoral® can form a microemulsion in the gastrointestinal tract after oral administration. However, long‐term use of large dose surfactants could easily cause mucosal damage and even gastrointestinal bleeding.^18^, ^19^, ^20^ This study fabricated enteric MSNs loaded with ILs and CyA to improve oral bioavailability without surfactants. This oral delivery system could further foster the application of ILs in pharmaceutical field.

## 2 MATERIALS AND METHODS

### 2.1 Materials

CyA was supplied from Sichuan Industrial Institute of Antibiotics (Chengdu, China). Sorbic acid, sodium fluoride, methanol, ethanol, phosphoric acid, hydrochloric acid, sodium hydroxide, and sodium dodecyl sulfate (SDS) were purchased from Sinopharm Chemical Reagent Co., Ltd. (Shanghai, China). Choline bicarbonate was obtained from Sigma‐Aldrich Co., Ltd. (Missouri, USA). Tetraethyl orthosilicate (TEOS) and cetyl trimethyl ammonium bromide (CTAB) were provided by Shanghai Aladdin Biochemical Technology Co., Ltd. (Shanghai, China). Eudragit® L100 was obtained from Evonik Industries AG (Essen, Germany). Neoral® was produced by Novartis Pharma Schweiz AG (Basel, Switzerland). Cyclosporine D (CyD) standard substance was obtained from Meilun Biotechnology Co., Ltd. (Shanghai, China). Rat gastric‐soluble capsules (size 9) with length maximum of 8.4 mm and external diameter maximum of 2.71 mm are obtained from Yuyan Instruments Co., Ltd. (Shanghai, China). Deionized water was prepared by a Milli‐Q purification instrument (Billerica, USA). All other reagents were of analytical grade.

### 2.2 Preparation and characterization of MSNs

The preparation of MSNs was based on the template method reported previously.^21^, ^22^ Briefly, 960 ml of deionized water was added into a 2 L round‐bottom flask and stirred mechanically at 200 rpm. Then 2.0 g of CTAB was added into the flask, followed by the addition of 7.0 ml of 2 mol/L NaOH solution. The reaction mixture was heated to 80°C and kept at 80°C for 30 min. Next, 14.0 ml of TEOS was added dropwise and the stirring speed was increased to 300 rpm. The mixture was allowed to react for 2 h. After reaction was finished, the reaction solution was cooled to room temperature and centrifuged at 20,000 g for 10 min to settle down the particles. Deionized water and ethanol were used to wash precipitates alternately twice, after which the particles were placed in a vacuum oven at 60°C and dried overnight. Reflux of 2.0 g dried MSNs in a mixed solution of 400 ml ethanol and 20 ml hydrochloric acid at 80°C for 36 h was used to remove template. After reflux, the recovered particles were washed alternately with deionized water and ethanol twice. Finally, particles were placed in a vacuum oven and dried at 60°C overnight.

After MSNs were dispersed in pure water at 25°C, particle size, polydispersity index (PDI), and zeta potential were measured by a Malvern Zetasizer Nano ZS analyzer system (Malvern Instruments Ltd., Malvern, UK). MSNs were also characterized by a Nicolet Fourier transform infrared spectrometer (FTIR, Thermo Fisher Scientific Inc., Waltham, USA) and a Tecnai G2 transmission electron microscope (TEM, FEI, Netherlands).

### 2.3 Preparation and characterization of choline sorbate ILs

Sorbate ILs (SCILs) were synthesized by a salt metathesis reaction. In brief, sorbic acid and choline bicarbonate (molar ratio of 1:1) were weighed separately. Then, sorbic acid was dissolved in an appropriate amount of methanol. Choline bicarbonate was put into a 100 ml round‐bottom flask. The methanol solution of sorbic acid was added dropwise into choline bicarbonate. Reaction was kept under magnetic stirring until no bubbles overflow. Next, solvents of the resultant mixture were removed by rotary evaporation at 60°C for 30 min. Finally, the synthesized SCILs were placed in a vacuum oven and dried for 48 h to get rid of residual solvents.

The SCILs were characterized by ^1^H‐nuclear magnetic resonance (^1^H‐NMR) in DMSO‐*d*_6_ with the residual solvent signals as the internal standard on a Bruker Ascend™ 600 MHz spectrometer (BRUKER AXS GmbH, Berne, Switzerland). The FTIR and differential scanning calorimetry (DSC) spectra of SCILs were recorded using a Nicolet FTIR spectrometer (Thermo Fisher Scientific Inc) and a 214 Polyma differential scanning calorimeter (NETZSCH Gerätebau GmbH, Selb, Germany), respectively.

### 2.4 Solubilization of CyA in SCILs

The saturated solubility of CyA was measured by dissolving excess CyA in solvent. In brief, excess CyA and 10 ml of methanol were dissolved in SCILs, then dried by rotary evaporation at 60°C for 30 min and underwent vacuum drying for 12 h to remove methanol. Subsequently, the solution was vortexed for 5 min and kept at 37°C ± 0.5°C for 2 h. Afterward, the mixture was subject to centrifugation two times for sampling. The first round of centrifugation at 4000 g, 10 min was intended to take samples from the bottom layer due to larger density of SCILs than CyA. The second round of centrifugation at 13,000 rpm for 10 min aimed at taking samples from supernatant to ensure no CyA precipitate contamination. The supernatant was followed by appropriate dilution with methanol. Twenty microliters were analyzed by high performance liquid chromatography (HPLC) to determine the saturated solubility of CyA in SCILs. Detail quantification method of CyA in methanol by HPLC was described in the supporting material S1.

The saturated solution of CyA in SCILs was diluted by adding deionized water to achieve weight ratio of 10/1, 10/2, 10/5, 10/10, 10/40, and 10/90 (SCILs/water). Afterward, the determination process of saturated solubility of CyA in SCILs diluted with water was the same as in SCILs.

### 2.5 Preparation and characterization of enteric MSNs co‐loading CyA and SCIL (CyA@SCIL@MSNs)

First, we used MSNs to load CyA and SCILs with different ratios, including CyA@SCIL@MSNs‐1 (0.05/0.2/1, w/w/w), CyA@SCIL@MSNs‐2 (0.05/0.5/1, w/w/w), CyA@SCIL@MSNs‐3 (0.05/1/1, w/w/w), and CyA@SCIL@MSNs‐4 (0.2/1/1, w/w/w). Briefly, MSNs, SCILs, and CyA were weighed according to the formulation ratios. Ultrasonication for 30 min was employed to promote the dissolution of CyA and SCILs. Then, the suspension was magnetically stirred at room temperature for 24 h to allow the CyA and SCILs fully to enter the MSNs channels. Subsequently, methanol was removed by rotary evaporation at 60°C for 30 min. The samples were dried in a vacuum oven at 60°C for 12 h to remove the residual solvents and stored in a desiccator for use. For enteric coating, the dried nanoparticles were coated by spraying 10% Eudragit® L100 solution (in methanol), then dried in a well‐ventilated place at room temperature for 4 h, and subsequently transferred to a vacuum oven at 60°C for 12 h to remove the residual solvents. The final products were ground until they could pass across 80‐mesh sieve. MSNs loading CyA was denoted as CyA@MSNs and used as control group. Enteric CyA@MSNs were prepared via the same method for enteric CyA@SCIL@MSNs but without the addition of SCILs.

The x‐ray powder (XRD) spectra of CyA, MSNs, CyA@MSNs, CyA@SCIL@MSNs, and enteric CyA@SCIL@MSNs were recorded in the 2*θ* range of 1^°^–50^°^ using x‐ray powder diffractometer (BRUKER AXS GmbH, Billerica, USA). A scanning electron microscope (SEM, Hitachi Regulus 8100, Japan) were used to characterize the morphology. Samples were dispersed on the conductive adhesive, sputtered with Au and observed under acceleration voltage of 3–15 kV.

### 2.6 In vitro release profiles

The in vitro release was evaluated using a 708‐DS dissolution tester (Agilent Technologies Inc.). For release of MSNs co‐loading CyA and SCILs with different ratios, the SDS aqueous solution (0.1%, 1000 ml) was used as dissolution media to evaluate the differences among various formulations well. The rotation speed was 75 rpm and the temperature was set at 37°C. At sampling timepoints of 5, 10, 15, 20, 30, 45, 60, 90, and 120 min, 1.5 ml of samples was taken out and equal volume of fresh dissolution media was supplemented immediately. The withdrawn samples were filtered through 0.22 μm polyether sulfones membrane and analyzed by HPLC for CyA. Detail quantification method of CyA in dissolution media by HPLC was described in the supporting material S1. After enteric coating, the leakage of CyA of enteric MSNs was investigated in pH 1.0 HCl (containing 0.1% SDS). The release behavior of CyA and SCILs from the enteric CyA@SCIL@MSNs in pH 1.0 hydrochloric acid (containing 0.1% SDS) and pH 6.8 phosphate buffer (containing 0.1% SDS) was investigated using the same dissolution method as above. The concentration of SCILs was represented by determination of sorbic acid, whose HPLC analysis method and validation were described in supporting material (Table S1–S7).

### 2.7 Pharmacokinetic study

In vivo pharmacokinetic studies were conducted in male Sprague–Dawley rats (Slac Laboratory Animal Co., Ltd., Shanghai, China). The protocol was approved by the Animal Ethics Committee of Fudan University. Before the experiment, all rats were kept in cages with controlled temperature (23 ± 1°C), relative humidity (50% ± 20%) and 12 h of light/dark cycle. They were allowed free access to standardized food and water and adapted for at least 5 days. Prior to pharmacokinetic study, rats were fasted for 12 h.

Thirty SD rats weighing 200–250 g were randomly divided into five groups (six rats per group). Enteric CyA@SCIL@MSNs‐4, nonenteric CyA@SCIL@MSNs‐4, enteric CyA@MSNs, CyA@SCIL and Neoral® was administered by gavage at an equivalent dose of CyA (20 mg/kg), respectively. CyA@SCIL (0.2/1, w/w) was fabricated by dissolving CyA in SCIL. CyA@SCIL was then encapsulated in gastric‐soluble rat capsules. The CyA‐SCIL capsules were prepared fresh before use to avoid any possible compromise of capsule shells. For Neoral® group, the content of Neoral® was dispersed in water as the final dosage form.

After intragastric administration, 0.5 ml of rat whole blood was taken from the orbital venous plexus and put into a centrifuge tube with heparin sodium. The blood sampling time intervals for CyA@SCIL group and Neoral® group were 0.5, 1, 1.5, 2, 2.5, 3, 4, 6, 8, 12, and 24 h, while for the other three groups, time intervals were set at 0.5, 1, 2, 3, 4, 5, 6, 7, 8, 10, 12, and 24 h. The sample treatment and analysis method of CyA in rat whole blood was described in supporting material S1.

### 2.8 Data analysis

The data were expressed as mean ± standard deviation (SD). For group comparison, one‐way analysis of variance (ANOVA) (Graphpad Prism 7.0 Software, LaJolla, CA, USA) was applied. When the *p* value was less than 0.05, the difference was considered statistically significant. The noncompartment model was applied during pharmacokinetics analysis for each animal via DAS software (version 2.0, Mathematical Pharmacology Professional Committee of China, Shanghai, China). The relative bioavailability (*RBA*) was calculated according to the following equation, using Neoral® group as the reference.

$$\mathrm{RBA}\left(\%\right)=\frac{{\mathrm{AUC}}_{\text{Test}}}{{\mathrm{AUC}}_{{\text{Neoral}}^{®}}}*100\%$$

## 3 RESULTS

### 3.1 Preparation and characterization of MSNs

**FIGURE 1:**
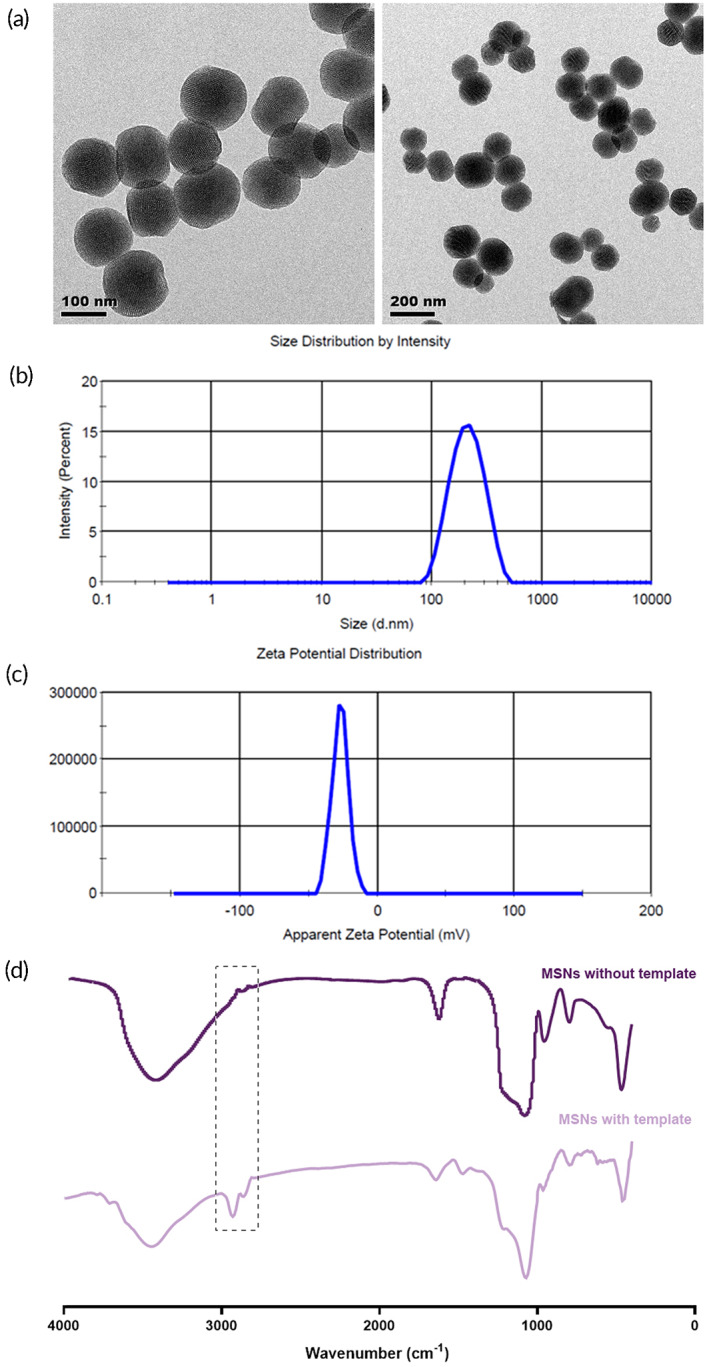
Characterization of mesoporous silica nanoparticles (MSNs). (a) Transmission electron microscope (TEM) images of MSNs, scale bar: 100 and 200 nm, respectively. (b) Size distribution of MSNs (*n* = 3). (c) Zeta potential of MSNs (*n* = 3). (d) Fourier transform infrared (FTIR) spectra of MSNs with and without template

MSNs were successfully prepared by template method reported previously.^21^, ^22^ MSNs showed a spherical‐like morphology with a size of ~200 nm, as characterized by TEM (Figure 1a). The particle size measured by dynamic light scattering method (Figure 1b) is (195.7 ± 10.2) nm (PDI = 0.152). The negative zeta potential ([−25.1 ± 4.32] mV, shown in Figure 1c) deriving from Si—OH group on the surface of MSNs evidenced the removal of template, otherwise the zeta potential would be positive, which was endowed by the remnant of template. Disappearance of a carbon chain band in the wavenumber range of 2700–3000 cm^−1^ indicated no template agent left in the channel of MSNs (Figure 1d), as well.

### 3.2 Preparation and characterization of SCILs

**FIGURE 2:**
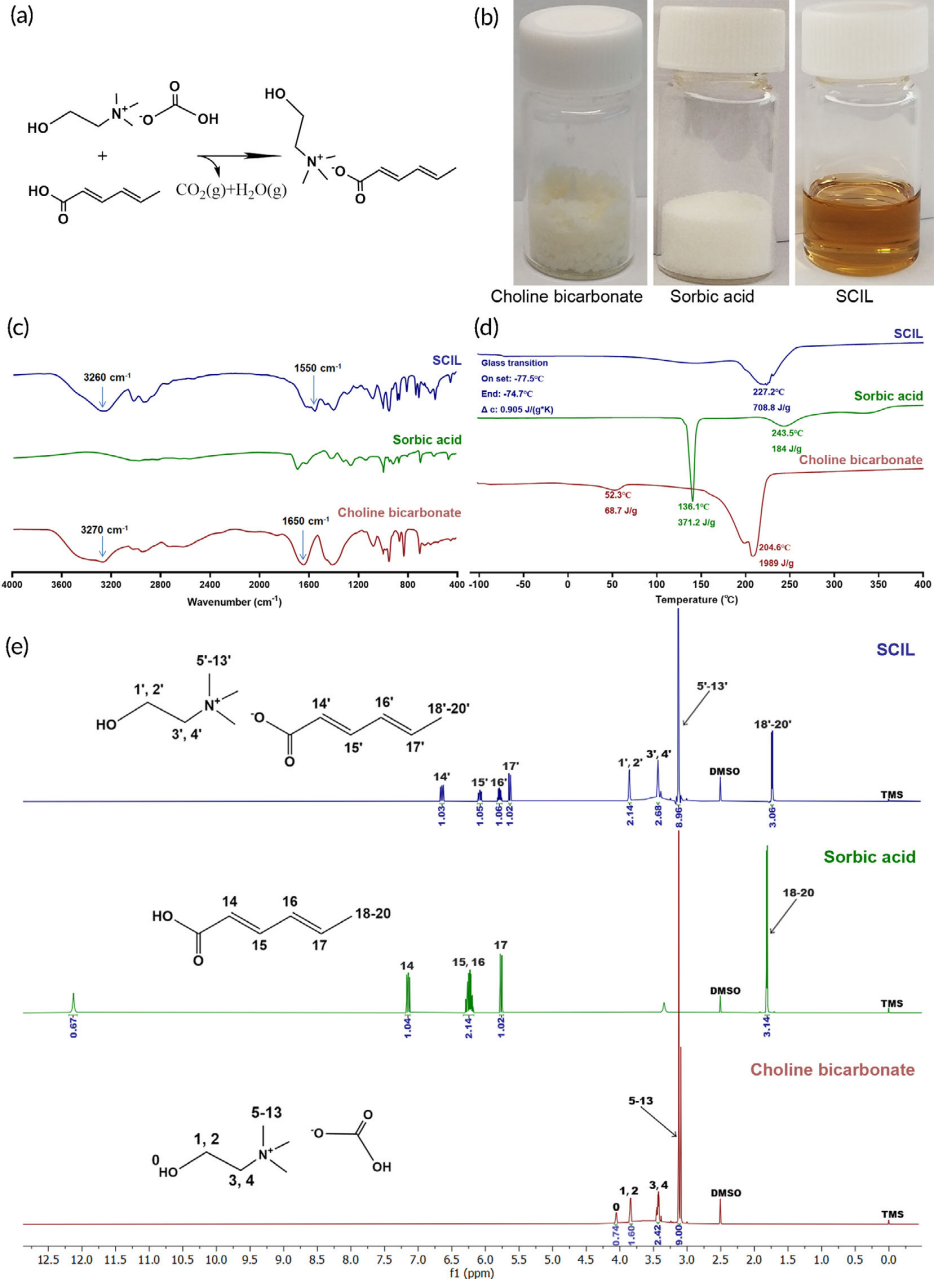
Preparation and characterization of sorbate ILs (SCIL). (a) Chemical reaction equation of SCILs synthesis. (b) Physical state of reactants and SCIL. (c) Fourier transform infrared (FTIR) spectra, (d) differential scanning calorimetry (DSC) spectra and (e) ^1^H‐NMR spectra of choline bicarbonate, sorbic acid and SCIL respectively

We successfully prepared SCILs with sorbic acid and choline bicarbonate using a conventional metathesis reaction (Figure 2a). SCILs were light yellow‐brown clear liquids at room temperature with high viscosity (Figure 2b). The FTIR spectra of SCIL, sorbic acid, and choline bicarbonate are displayed in Figure 2c. The absorption peak of C=O in sorbic acid is located at 1690 cm^−1^, while in the generated SCILs, the absorption peak of C=O is located at 1550 cm^−1^. Regarding —OH group, the absorption peak changes from 3270 cm^−1^ in choline bicarbonate to 3260 cm^−1^ in generated SCILs. The distinct movement of the C=O absorption band evidenced the formation of ionic bonds in SCIL. The DSC spectra (Figure 2d) show that the glass transition temperature (−77.5 to 74.7°C) of the synthesized SCIL was lower than the melting point (the former endothermic peak in DSC spectra) of any synthetic raw materials. The latter endothermic peak of choline bicarb and sorbic acid is supposed to be decomposition peak. The ^1^H‐NMR spectra of SCIL, sorbic acid, and choline bicarbonate are shown in Figure 2e. Take SCIL as an example, its NMR chemical shift is ^1^H‐NMR (600 MHz, DMSO‐*d*_6_) *δ* 6.65 (dd, *J* = 15.0, 10.9 Hz, 1H), 6.08 (t, *J* = 13.2 Hz, 1H), 5.79 (m, 1H), 5.64 (d, *J* = 15.2 Hz, 1H), 3.87 (dt, *J* = 9.0, 2.8 Hz, 1H), 3.85 (dt, *J* = 9.0, 3.0 Hz, 1H), 3.44 (t, *J* = 4.8 Hz, 3H), 3.13 (s, 9H), 1.74 (d, *J* = 6.8 Hz, 3H). Comparing the ^1^H‐NMR spectra of the raw materials with SCIL, it can be found that the chemical shift of each proton on the choline fragment had almost no change after the reaction; the proton peak of carboxylic acid groups of sorbic acid disappeared, and the rest proton peaks that affected by newly‐generated ionic bonds moved to the upfield. Notably, the chemical shift of 14‐H moved from *δ* 7.15 to *δ* 6.65 ppm, exhibiting a change of 0.5 ppm, which is also a strong proof of the successful synthesis of SCIL. In addition, the water content of neat IL determined by Karl Fischer titration was 1.73% ± 0.26% (see supporting material S1).

### 3.3 Solubilization of CyA in diluted SCILs

**FIGURE 3:**
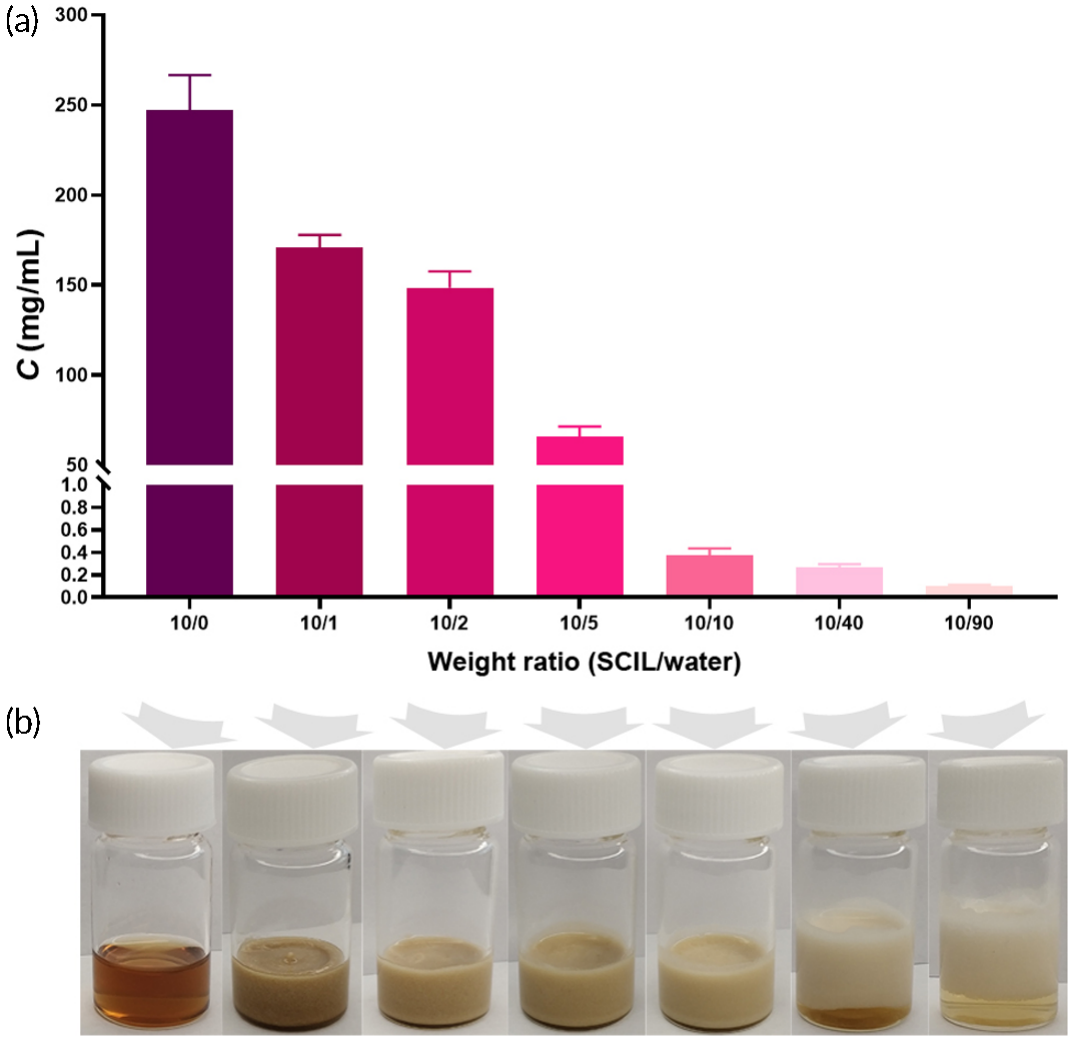
Saturated solubility of cyclosporine A (CyA) (a) and physical states (b) in sorbate ILs (SCILs) with different percentages of water (*n* = 3)

Figure 3 illustrated that the saturated solubility of CyA in SCILs had almost reached 250 mg/ml. However, the solubility of CyA decreased sharply when SCILs was diluted by water. It was obvious that a large amount of CyA precipitated out in diluted SCILs, making the SCILs solution turbid. When SCILs were diluted with the same weight of water or more (≥10:10), the saturated solubility of CyA in the solution was even less than 1 mg/ml. Considering the absence of pH‐dependent saturated solubility of CyA in different pH aqueous media (Table S9), this phenomenon indicated that if CyA@SCIL were directly administered orally, a large amount of CyA would precipitate out in the stomach. Thus, we cannot leverage the delivery advantage of SCILs, highlighting the need for suitable preparation methods to avoid the unfavorable precipitation of CyA in the gastric juice.

### 3.4 Preparation and characterization of enteric CyA@SCIL@MSNs

**FIGURE 4:**
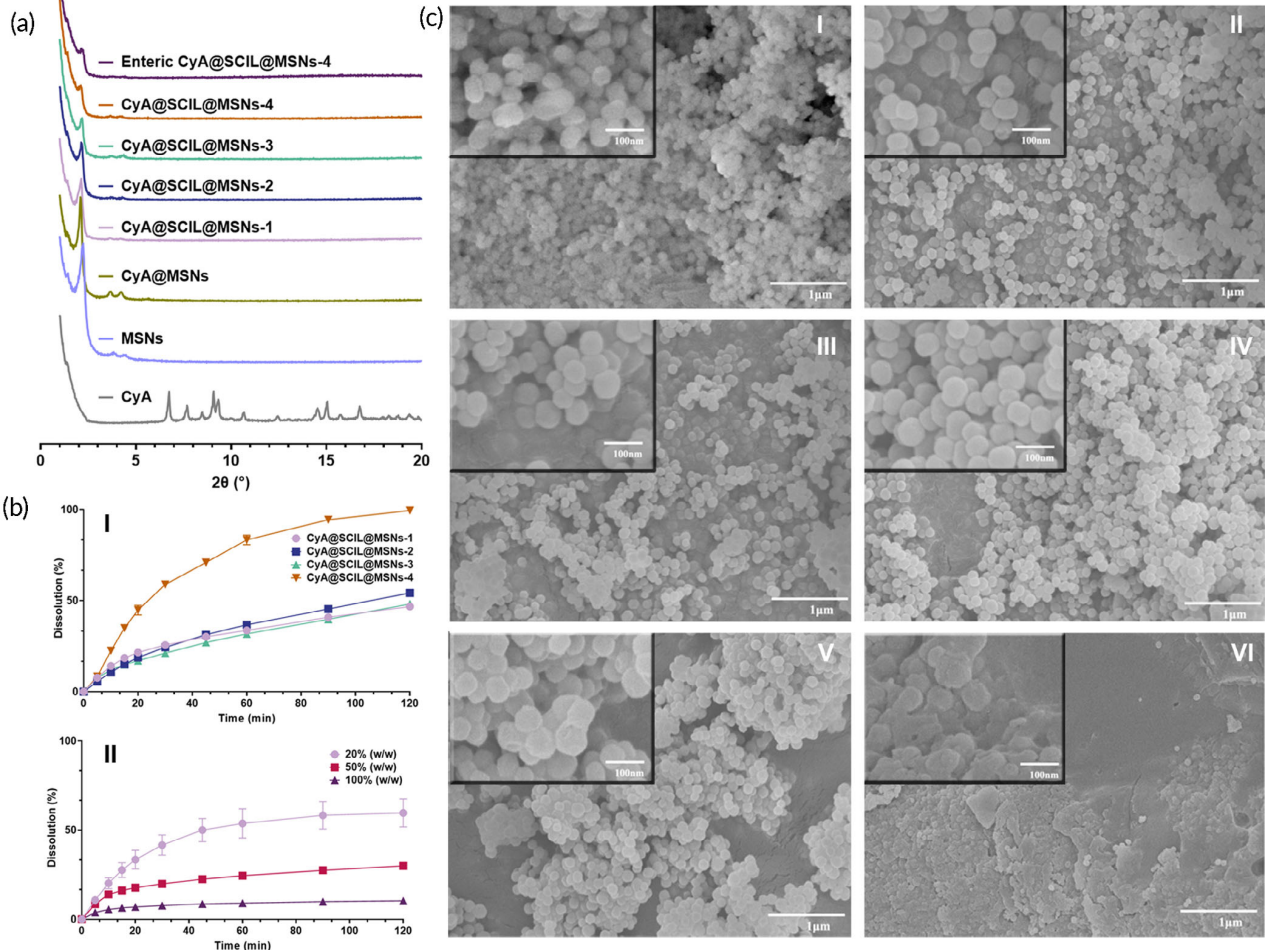
Characterization of enteric mesoporous silica nanoparticles (MSNs) co‐loading cyclosporin A (CyA) and sorbate ionic liquids (SCIL). (a) XRD spectra of CyA, MSNs, CyA@MSNs, CyA@SCIL@MSNs, and enteric CyA@SCIL@MSNs. (b) Release curves of CyA from MSNs with different amounts of CyA and SCILs in 0.1% sodium dodecyl sulfate (SDS) aqueous solution (I) and leakage of CyA from MSNs with different coating weight gain in pH 1.0 HCl (containing 0.1% SDS) (II) (*n* = 2). (c) SEM images of MSNs (I), CyA@SCIL@MSNs‐1 (II), CyA@SCIL@MSNs‐2 (III), CyA@SCIL@MSNs‐3 (IV), CyA@SCIL@MSNs‐4 (V) and enteric CyA@SCIL@MSNs‐4 (VI)

CyA@SCIL@MSNs were prepared by adsorbing CyA@SCIL into MSNs. Given the high viscosity of neat ILs, the dissolving rate and diffusion rate of CyA are extremely slow within SCILs. Thus, CyA could not well dispersed in SCILs to form a homogenous solution in a short time. To address this problem, we first dissolved SCILs and CyA in methanol to achieve a well‐mixed solution. Next, we suspended MSNs in an appropriate amount of methanol mixture. Methanol was subsequently removed by rotary evaporation at 60°C for 30 min and vacuum drying at 60°C for 12 h. Eudragit® L100 was used as enteric materials to coat the MSNs. As shown in Figure 4a, the characteristic peak of raw CyA could not be observed in the diffractograms of MSNs loaded with CyA, indicating the transformation of crystal CyA into amorphous CyA and successful loading of CyA into the pores of MSNs. By comparing different CyA@SCIL@MSNs formulations, it also could be discovered that as the dosage of SCILs increased, the characteristic peak of MSNs (2*θ* ≈ 2°) gradually reduced, indicating that the pores of MSNs were gradually filled. XRD diffractograms proved that it was practicable to reasonably increase CyA and SCILs loading of MSNs.

Dissolution of four nonenteric CyA@SCIL@MSNs in 0.1% SDS aqueous solution are shown in Figure 4b,I. The results showed that the increase in the proportion of SCILs had little impact on the dissolution rate. When the CyA@SCIL@MSNs formulation ratio was 0.2/1/1, the dissolution endpoint appeared within 2 h. Thus, CyA@SCIL@MSNs‐4 was deemed as the best formulation and used in follow‐up experiments. The coating weight gain affected leakage of CyA in pH 1.0 HCl significantly. Dissolution results after coating (Figure 4b,II) indicated that with the increase of Eudragit® L100, the cumulative release amount of CyA in the pH 1.0 HCl (containing 0.1% SDS) decreased. Therefore, the coating weight gain was set at 100%.

Compared with Figure 4c,I, despite the increased amount of SCILs and CyA in MSNs, the surface morphology of MSNs had not changed (Figure 4c,II–V), which meant that SCILs and CyA had been incorporated into the MSNs channels. In addition, it could be found from Figure 4c,VI that the coating agent was evenly coated on the surface of the MSNs.

What is more, after 10 months storage, the CyA content in enteric CyA@SCIL@MSNs‐4 and the dissolution curves of CyA from enteric CyA@SCIL@MSNs‐4 were close to those at Day 0, demonstrating the robust stability after long‐term storage (Figure S3).

### 3.5 In vitro release profiles

**FIGURE 5:**
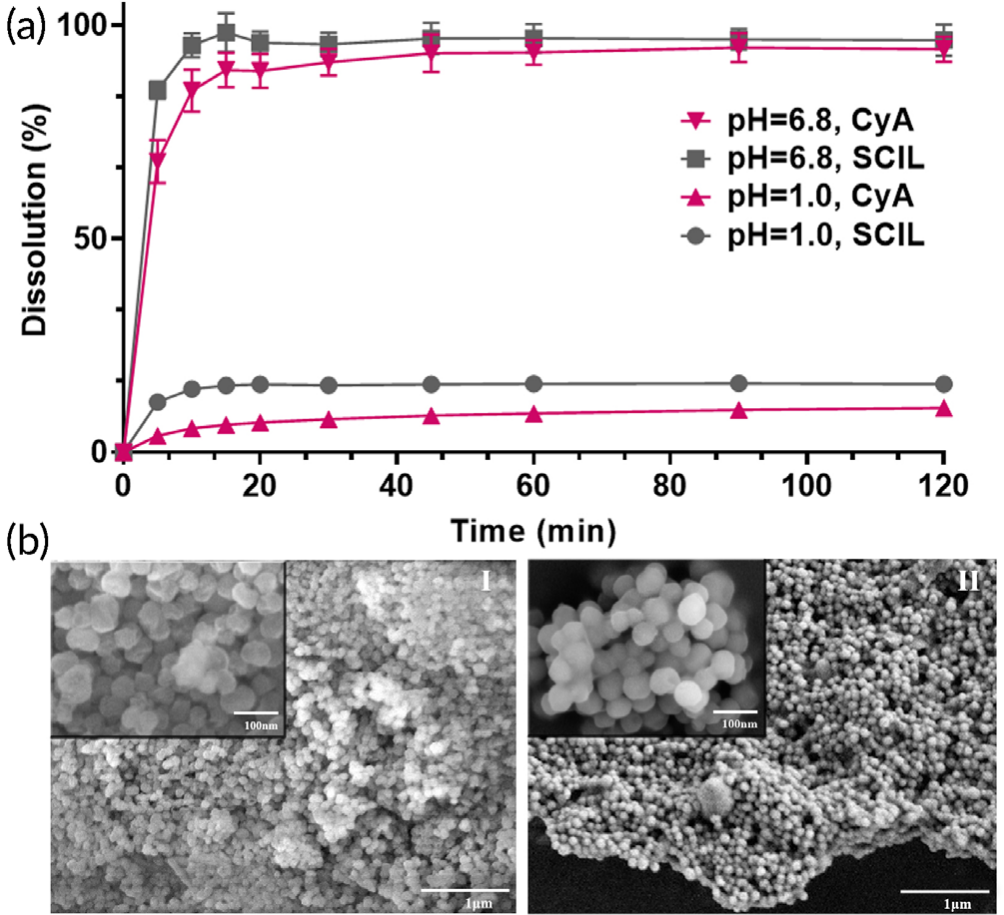
In vitro release of enteric CyA@SCIL@MSNs‐4. (a) Dissolution profiles of cyclosporin A (CyA) and sorbate ionic liquids (SCIL) from enteric CyA@SCIL@MSNs‐4 in pH 1.0 hydrochloric acid (containing 0.1% sodium dodecyl sulfate [SDS]) and pH 6.8 phosphate buffer (containing 0.1% SDS) (*n* = 3). (b) Scanning electron microscope (SEM) images of the residue after dissolution of the enteric CyA@SCIL@MSNs‐4 in pH 6.8 phosphate buffer (containing 0.1% SDS) (I) and in pH 1.0 hydrochloric acid (containing 0.1% SDS) (II)

The release profiles of CyA and SCILs (Figure 5a) showed that only 10% of both CyA and SCILs were released due to the protection of enteric coating in pH 1.0 HCl. However, more than 85% of CyA and SCILs could be released within 15 min in pH 6.8 phosphate buffer. Moreover, the release of CyA and SCILs was synchronized in pH 6.8 phosphate buffer. After destruction of enteric coating in pH 6.8 phosphate buffer, the fine morphology of original MSNs still could be observed (Figure 5b,I), indicating that the nano‐scale MSNs could be uniformly dispersed as a secondary drug release unit after the enteric coating was destroyed, and payload could be released from pores. As shown in Figure 5b,II inset, the aggregation and adhesion caused by enteric membrane could be observed, indicating enteric coating membrane was not be destroyed in pH 1.0. This proved that translation of ILs to be enteric nanoparticles was feasible.

### 3.6 Pharmacokinetic study

**FIGURE 6:**
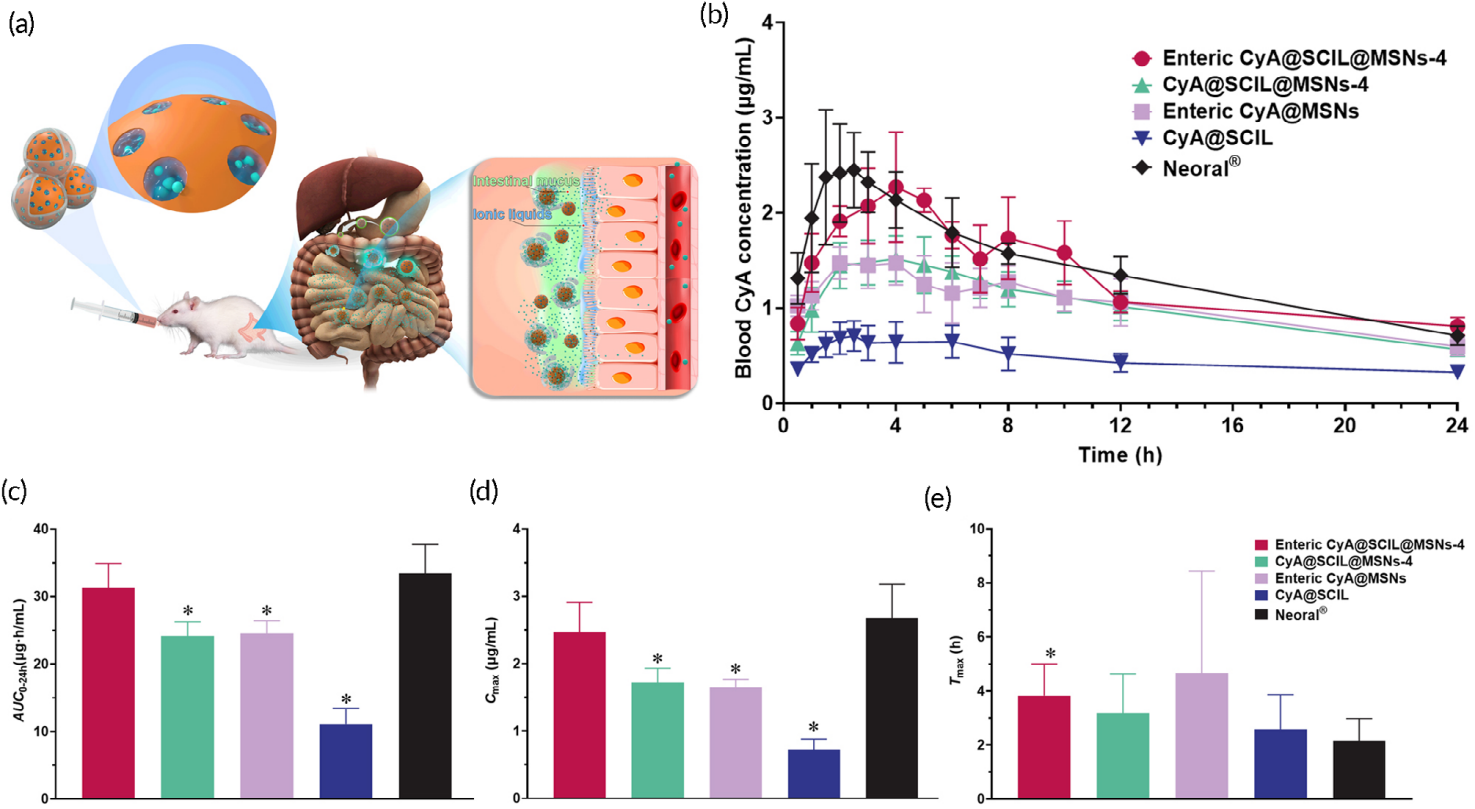
Pharmacokinetic study of various cyclosporin A (CyA) formulations. (a) Schematic illustration of the protection capability of enteric coating and enhanced permeation of CyA assisted by sorbate ILs (SCIL) in vivo after oral administration to rats. (b) The concentration–time curves of CyA after oral administration of a 20 mg/kg single dose of enteric CyA@SCIL@MSNs‐4, CyA@SCIL@MSNs‐4, enteric CyA@MSNs, CyA@SCIL and Neoral® in rats. (c–e) Comparison of pharmacokinetic parameters, (c) AUC_0–24 h_, (d) *C*_max_ and (e) *T*_max_ (*n* = 6, *p < 0.05 compared with Neoral®)

After oral administration, the concentration–time curves of CyA are shown in Figure 6. It could be concluded that the concentration of CyA in Neoral® group reached the peak at the most rapid rate and the peak concentration was the highest among five groups. There was a double‐peak phenomenon in the concentration–time curve of enteric formulations (enteric CyA@SCIL@MSNs‐4 and enteric CyA@MSNs), which could be caused by polydispersity of enteric nanoparticles in gastrointestinal tract. The required time for enteric nanoparticles to reach the absorption sites is varying. The release and absorption of CyA from some enteric nanoparticles that reach the absorption sites more slowly overlapped with the concentration of CyA previously absorbed into the blood, inducing a double peak. The pharmacokinetic parameters AUC, *C*_max_, and *T*_max_ were statistically analyzed (Figure 6c–d). The results showed that the AUC_0–24 h_ of enteric CyA@SCIL@MSNs‐4 was significantly different from other three control formulations except Neoral®. There was no significant difference of AUC_0–24 h_ between enteric CyA@SCIL@MSNs‐4 and Neoral®. However, the AUC_0–24 h_ of other three control formulations was significantly lower than Neoral®. This is also true for *C*_max_. Conversely, the *T*_max_ of enteric CyA@SCIL@MSNs‐4 was not significantly different from other three control formulations except Neoral®. Yet, there was no significant difference of *T*_max_ between other three control formulations and Neoral®. The *T*_max_ of Neoral® was basically the same as reported in the relevant literature.^23^ Release site of enteric CyA@SCIL@MSNs‐4 was at the small intestine, thus the *T*_max_ was relatively longer. We also found that compared with CyA@SCIL, the enteric CyA@MSNs could also improve the oral bioavailability of CyA.

## 4 DISCUSSION

Oral delivery is a common administration route with high patient compliance. However, a lot of drug molecules are not suitable to be delivered by oral route due to their low aqueous solubility and membrane permeability,^24^ such as BCS II and IV drugs. Hence, more and more novel technologies are emerging for improving oral bioavailability of these drugs, including solid dispersion,^25^ self‐microemulsion,^26^ nanoparticles,^27^ and permeation enhancers.^28^ These technologies must be combined with traditional formulations to meet requirements of clinical administration. For instance, self‐microemulsions are filled in soft capsules^29^ and solid dispersions are prepared to be tablet after mixing with other excipients.^30^

ILs have been widely used as solvents or permeation enhancers to dissolve poorly soluble drugs^31^ or enhance transmembrane ability of drug molecules.^32^ In addition, they have been reported to enhance oral absorption of insulin^33^ and poorly soluble drugs.^34^ ILs can be tailored to have different physiochemical properties by pairing different ions. In this study, we employed sorbic acid and choline to form SCILs. Both sorbic acid and choline are recognized by the Food and Drug Administration (FDA) as generally regarded as safe (GRAS) ingredients.^35^, ^36^ Sorbic acid is a monoacid that reacts with choline bicarbonate to form ionic bond. The reaction products are liquid viscous state with brown color. The strong ionic bonds and hydrogen bonds break the crystal structure, which results in ILs. ILs are able to dissolve the CyA with high solubility (250 mg/ml). However, CyA separated out as diluted by water, which indicated that the solubility of CyA was decreased dramatically in mixed solution of SCILs and CyA. We also found that the extent of solubility decrease was related to organic acids of ILs (Figure S1, S2 and Table S8). In other words, different ILs have varied resistance ability to water dilution. ILs were dissociated as anions and cations in water because they are actually salts. However, the structure of ILs cannot be broken completely when a small amount of water was added. The involvement of water could form new structure with ILs.^9^ When 50% water existed, numerous CyA separated out and the solubility was decreased to 0.3 mg/ml, which implies most of SCILs were dissociated and lost their solubilization capacity. This in vitro test supported our hypothesis that ILs could not exert their effect well if they were administered orally without any protection. Thus, most study adopted intrajejunal administration to elucidate their absorption‐enhancing effect.^33^ It is necessary to develop a formulation strategy, which is suitable for clinical translation to protect ILs against dissociation.

Although some studies put ILs in enteric soft capsules for oral administration, ILs could destroy the capsule during long‐term storage due to their strong solubilization capacity. Hence, it is preferred that ILs are encapsulated in enteric micro/nano‐particles and distributed in small intestine in large area. This study employed MSNs to carry ILs for overcoming these drawbacks of ILs in oral delivery. MSNs have a lot of pores, which are able to adsorb a large volume of liquids^37^ and MSNs have been widely used in pharmaceutical excipients due to good biocompatibility.^38^ MSNs can carry 50% SCILs in their pores and CyA is dissolved in SCILs. The loading of CyA can controlled by its concentration in SCILs. The results showed the loading efficiency of CyA and SCILs in MSNs had no any influence on release of CyA. Hence, we selected CyA@SCIL@MSNs‐4 (0.2:1:1, w/w/w) for subsequent studies because it is suitable for animal dosing. After loading SCILs in MSNs, MSNs still look like solid state with good fluidity. In order to protect SCILs through stomach, we used Eudragit® L100 as enteric materials to coat MSNs. Eudragit® L100 is a common enteric material, which is dissolved in circumstances over pH 6.0.^39^ The leakage of CyA in pH 1.0 media was about 10% when coating weight gain was 100% and the leakage increased as the coating weight gain decreased. Nanoparticles are very difficult to be coated completely due to their extremely small size. Hence, we finally used enteric nanoparticles with coating weight gain of 100% for animal study. Some enteric nanoparticles could be aggregated by binding with coating materials; however, they were dispersed as original nanoparticles in pH 6.8 release media as detected by SEM.

As permeation enhancer, it is critical that it should reach absorption site together with drugs simultaneously. Hence, the release rate of SCILs should be similar with that of drugs (CyA). Because SCILs would dissociate in media after release, we represented the concentration of SCILs by determining the sorbic acid. The results showed SCILs and CyA released from MSNs in both pH 1.0 and pH 6.8 media synchronously. After oral administration of enteric nanoparticles, they passed through stomach to small intestine. Nanoparticles would distribute everywhere with the movement of small intestine and could penetrate mucus layer due to their small size.^40^ After nanoparticles enter mucus layer, the enteric coats dissolved gradually due to suitable pH. Then, CyA and SCILs released from MSNs simultaneously, which enabled SCILs to exert their permeation‐enhancing capability well. Meanwhile, CyA is not easy to separate out in mucus due to the presence of SCILs. In addition, SCILs were distributed in large area by transportation of MSNs, which would not lead to local high concentration and severe mucus toxicity.

The oral bioavailability of enteric nanoparticles has no statistically difference with Neoral® after oral administration to rats, which demonstrated that the combination of SCILs and MSNs were able to improve the oral absorption of CyA, because Neoral® is the most successful formulation of CyA which employs self‐microemulsion to facilitate oral absorption of CyA.^41^ The bioavailability of nonenteric nanoparticles (CyA@SCIL@MSNs‐4) is lower than that of enteric nanoparticles significantly (Figure 6c). Although nonenteric nanoparticles also improved oral absorption of CyA to a certain extent, their effects were similar with the group without SCILs (enteric CyA@MSNs), which implies SCILs could release and dissociate in stomach immediately if nanoparticles were not protected by enteric materials. The in vivo study also confirmed that ILs could be dissociated in stomach and lost their capability of improving absorption, as supported by low oral bioavailability of CyA@SCIL group. The oral bioavailability of nanoparticles without SCILs (enteric CyA@MSNs) is also lower than that of enteric CyA@SCIL@MSNs significantly, implying the permeation‐enhanced absorption effect exerted by SCILs. Although the onset of therapeutic action of enteric CyA@SCIL@MSNs was slower than Neoral® due to enteric coating (Figure 6e), the bioavailability and *C*_max_ (Figure 6d) were not changed significantly. Consequently, ILs must avoid the dilution of gastric fluids as oral carriers or permeation enhancers. And ILs can be adsorbed into MSNs to achieve higher level of enhancing oral absorption. In addition, we evaluated the in vivo toxicity of Neoral® and enteric CyA@SCIL@MSNs by 7 days of once‐a‐day repeat oral administration to rats. The results showed though the rats body weight steadily increased in both groups, the weight gain of rats in the group of enteric CyA@SCIL@MSNs‐4 was significantly more than that of Neoral group (Figure S4), implying enteric CyA@SCIL@MSNs‐4 reduced irritation to intestinal tracts compared to Neoral®. Meanwhile, the extent of tissue inflammation induced by enteric CyA@SCIL@MSNs was lower than Neoral® (Figure S5). Besides, we only observed the irritation to GI tract after 7 days consecutive administration of two formulations, the difference of in vivo toxicity between two groups was not very large. It might be more evident if they are administered for long periods of time in clinical use.

## 5 CONCLUSION

Although ILs could be good permeation enhancer or solubilizer, some evident shortcomings limited their use in oral delivery, such as rapid drug precipitation when exposed to enormous gastric fluids This study employed MSNs to load ILs, which dissolved CyA and avoided leakage in stomach by enteric coating. This delivery system is able to protect ILs against dissociation in gastric fluids and ensure synchronous release of SCILs and CyA in small intestine. In addition, the enteric nanoparticles loading SCILs can improve the oral absorption of CyA significantly and achieve similar oral bioavailability with Neoral®. Except for BCS IV drugs, this system has great potential to be a platform for delivering other poorly soluble drugs and even biologics orally.

## AUTHOR CONTRIBUTIONS

**Kaiheng Liu:** Investigation (equal); methodology (equal); writing – original draft (equal). **Wenjuan Liu:** Investigation (equal); methodology (equal); writing – original draft (equal). **Zirong Dong:** Investigation (supporting). **Luyu Zhang:** Investigation (supporting). **Qiuyu Li:** Investigation (supporting). **Renjie Zhang:** Investigation (supporting). **Haisheng He:** Supervision (supporting). **Yi Lu:** Supervision (supporting). **Wei Wu:** Supervision (supporting). **Jianping Qi:** Conceptualization (lead); funding acquisition (lead); methodology (lead); project administration (lead); resources (lead); supervision (lead); writing – review and editing (lead).

### PEER REVIEW

The peer review history for this article is available at https://publons.com/publon/10.1002/btm2.10405.

## Supporting information


**Appendix S1** Supporting Information

## Data Availability

The data that support the findings of this study are available within the paper and its supplementary information. Any other data are available from the corresponding authors upon request.
