# Supplementary material for: Translation of ionic liquids to be enteric nanoparticles for facilitating oral absorption of cyclosporine A
Source: Bioeng Transl Med. 2022 Sep 7;8(2):e10405. doi: 10.1002/btm2.10405 (PMC10013816; doi:10.1002/btm2.10405)
Supplement: Supplementary file 1 — Appendix S1 Supporting Information [file BTM2-8-e10405-s001.docx]

Supporting Material

**Translation of ionic liquids to be enteric nanoparticles for facilitating oral absorption of cyclosporine A**

Kaiheng Liu^#^, Wenjuan Liu^#^, Zirong Dong, Luyu Zhang, Qiuyu Li, Renjie Zhang, Haisheng He, Yi Lu, Wei Wu, Jianping Qi*

Key Laboratory of Smart Drug Delivery of MOE, School of Pharmacy, Fudan University, Shanghai 201203, China

*Corresponding author: Jianping Qi, [qijianping@fudan.edu.cn](mailto:qijianping@fudan.edu.cn).

^#^These authors contributed equally.

**1. Methods**

- 1. **Quantification of CyA in methanol and dissolution media by HPLC**

The Agilent Technologies 1260 HPLC (Agilent, Santa Clara, USA) system was used with an ultraviolet detector set at wavelength of 210 nm and the column heater set at 70°C. The autosampler injection volume was 20 μL. Separation was performed on an Agilent ZORBAX StableBond 300 C18 Column (4.6 x 150 mm, 5 µm) with an Agilent ZORBAX StableBond 300 C18 (4.6 x 12.5 mm, 5 µm) guard column. Methanol-acetonitrile-0.2% aqueous phosphoric acid (5:70:25, v/v) solution mixture was chosen as the mobile phase and the flow rate was set at 1.5 mL/min. The chromatographic data was evaluated by the Agilent chromatographic workstation.

**1.2 Quantification of sorbic acid in two dissolution media by HPLC**

The Agilent Technologies 1260 HPLC (Agilent, Santa Clara, USA) system was used with an ultraviolet detector set at wavelength of 242 nm and the column heater set at 40°C. The autosampler injection volume was 20 μL. Separation was performed on an Agilent ZORBAX StableBond 300 C18 Column (4.6 x 150 mm, 5 µm) with an Agilent ZORBAX StableBond 300 C18 (4.6 x 12.5 mm, 5 µm) guard column. Acetonitrile-0.2% aqueous phosphoric acid (50:50, v/v) solution mixture was chosen as the mobile phase and the flow rate was set at 1.0 mL/min. The chromatographic data was evaluated by the Agilent chromatographic workstation.

- 1. **Quantification of CyA in rat whole blood by HPLC**

**1.3.1 Sample treatment**

After sampling, 400 μL of whole blood was added to a 5 mL centrifuge tube and mixed with 20 μL of internal standard (CyD, 50 μg/mL) and 15 mg of sodium fluoride under vortex mixing for 30 s. Then 3 mL of ether was added and vortexed for 7 min. The upper organic layer was taken and dried under nitrogen flow at 40℃ after centrifugation at 13000 rpm for 10 min. Next, 200 μL of methanol-0.05 mol/L HCl solution (6: 4, v/v) and 400 μL of n-hexane were added and vortex mixed for 3 min. The suspension was centrifuged again at 13000 rpm for 10 min and 50 μL of the lower layer was analyzed to obtain Cy A concentration.

**1.3.2** **Instrumentation and chromatographic condition**

The Agilent 1260 liquid chromatography (Agilent, Santa Clara, USA) was used to determine CyA in rat whole blood samples. The chromatographic detection wavelength was 210 nm. Agilent ZORBAX StableBond 300 C18 (4.6 x 150 mm, 5 µm) and Agilent ZORBAX StableBond 300 C18 (4.6 x 12.5 mm, 5 µm) guard column were for separation. The column temperature was set to 70 ℃. Methanol-acetonitrile-0.2% phosphoric acid water (4: 58: 38, v/v) was used as the mobile phase with flow rate at 1.5 mL/min and the injection volume of 50 μL.

- 1. **Preparation and characterization of different ionic liquids.**
     1. **Preparation and characterization of choline malate ionic liquids (MCILs)**

Malic acid and choline bicarbonate (molar ratio of 1:2) were weighed separately. Then, malic acid was dissolved in an appropriate amount of methanol. Choline bicarbonate was put into a 100 mL round-bottom flask. The methanol solution of malic acid was added dropwise into choline bicarbonate. Reaction was kept under magnetic stirring until no bubbles overflow. Next, solvents of the resultant mixture were removed by rotary evaporation at 60 °C for 30 min. Finally, the synthesized MCILs were placed in a vacuum oven and dried for 48 h to get rid of residual solvents.

The MCILs were characterized by ^1^H-NMR in DMSO‑d6 with the residual solvent signals as the internal standard on a Bruker Ascend^TM^ 600 MHz spectrometer (BRUKER AXS GmbH, Berne, Switzerland) (**Figure S1**).

- - 1. **Preparation and characterization of choline citrate ionic liquids (CCILs)**

Citric acid and choline bicarbonate (molar ratio of 1:3) were weighed separately. Then, citric acid was dissolved in an appropriate amount of methanol. Choline bicarbonate was put into a 100 mL round-bottom flask. The methanol solution of citric acid was added dropwise into choline bicarbonate. Reaction was kept under magnetic stirring until no bubbles overflow. Next, solvents of the resultant mixture were removed by rotary evaporation at 60 °C for 30 min. Finally, the synthesized CCILs were placed in a vacuum oven and dried for 48 h to get rid of residual solvents.

The CCILs were characterized by ^1^H-NMR in DMSO‑d6 with the residual solvent signals as the internal standard on a Bruker Ascend^TM^ 600 MHz spectrometer (BRUKER AXS GmbH, Berne, Switzerland) (**Figure S2**).

- 1. **Solubilization of CyA in different ionic liquids.**

The determination process of saturated solubility of CyA in MCILs and CCILs diluted or not with water were the same as in SCILs diluted or not with water (**Table S8**).

- 1. **Moisture content**

Moisture content of SCILs was determined by Karl Fischer titration using a volumetric Karl Fischer Titrator MKS-520 (Mettler Toledo, Columbus, Ohio USA). All measurements were conducted in triplicate. To perform the analysis, the instructions of the Standard Operating Procedure (SOP) of the company were followed.

- 1. **The stability of enteric CyA@SCIL@MSNs-4**

To investigate the stability of enteric CyA@SCIL@MSNs-4, CyA content in enteric CyA@SCIL@MSNs-4 and the dissolution curves of enteric CyA@SCIL@MSNs-4 after 10 months storage were determined in triplicate. Briefly, CyA@SCIL@MSNs-4 was sonicated in methanol to extract CyA. Then dissolved CyA in methanol was filtered, diluted to appropriate concentration, and subject to HPLC to determine CyA content. The dissolution profiles of enteric CyA@SCIL@MSNs-4 in pH 1.0 hydrochloric acid (containing 0.1% SDS) and pH 6.8 phosphate buffer (containing 0.1% SDS) during 2 h were determined.

- 1. **Oral biocompatibility *in vivo***

Male Sprague-Dawley rats, weighing (220 ± 10) g, were divided into two groups with 5 rats per group. One group was orally administrated with Neoral^®^ at a 20 mg/kg dose of CyA as control group, while the other group was administrated with enteric CyA@SCIL@MSNs-4 by gavage at the same dose of CyA. All groups were subject to 7 days of once-a-day repeat oral dosing. During the experiment period, the rats body weight was recorded daily and histological examination of intestine samples (jejunum, ileum, and colon) which were collected after 7 days consecutive dosing was conducted. Intestine tissues were fixed in 4% paraformaldehyde for 24 h, dehydrated in 70% ethanol, and then embedded in paraffin. The tissue sections were cut into 5 µm thickness, deparaffinized, rehydrated, and stained with hematoxylin and eosin (H&E). Histological morphology was visualized using a brightfield slide scanner microscope (Olympus VS 200, Tokyo, Japan). The severity of histological damage was analyzed in a blinded fashion to prevent observer bias.

**2. Results**

**2.1** **Quantification of CyA in methanol and dissolution media by HPLC**

CyA could be well separated from impurities with a retention time of 2.6 min. Linear equation, linear range, and correlation coefficient of CyA in methanol, pH=1.0 HCl (contain 0.1 % SDS), pH = 6.8 phosphate buffer (contain 0.1 % SDS) and 0.1 % SDS were shown in **Table S1**. The accuracy for samples of low, medium and high concentrations and the precision for within-day and between-day assays met the requirements of *in vitro* determination (shown in **Tables S2** and **Table S3**).

**Table S1** Linear equation, linear range, and correlation coefficient of CyA in methanol, pH=1.0 HCl (contain 0.1 % SDS), pH = 6.8 phosphate buffer (contain 0.1 % SDS) and 0.1 % SDS.

| **Media** | **Linear equation** | **r** | **Linear range (µg/mL)** |
| --- | --- | --- | --- |
| Methanol | *C** = 0.039 *A****^#^*** - 0.512 | 0.9998 | 0.099 ~ 198 |
| pH = 1.0 | *C* = 0.047 *A* + 0.402 | 0.9998 | 0.5025 **~** 40.2 |
| pH = 6.8 | *C* = 0.046 *A* + 0.218 | 0.9999 | 0.52 **~** 41.6 |
| 0.1 % SDS | *C* = 0.047 *A* + 0.255 | 0.9999 | 0.5125 **~** 41 |

**C* represents concentration, **^#^***A* represents peak area.

**Table S2** Accuracy of CyA determined by HPLC. (*n* = 3)

| C_added_ (μg/mL) | | | C_detected_ (μg/mL) | Recovery (%) | Mean ± SD (%) | RSD (%) |
| --- | --- | --- | --- | --- | --- | --- |
| Methanol | 0.99 | | 0.98 ± 0.02 | 98.99 | 98.79 ± 2.82 | 2.85 |
|  | 9.9 | | 9.56 ± 0.35 | 96.57 |  |  |
|  | 99 | | 99.39 ± 1.27 | 100.39 |  |  |
| pH = 1.0 | | 0.5025 | 0.52 ± 0.01 | 102.49 | 100.02 ± 2.15 | 2.15 |
|  |  | 5.025 | 4.98 ± 0.03 | 99.17 |  |  |
|  |  | 20.1 | 19.78 ± 0.13 | 98.39 |  |  |
| pH = 6.8 | | 0.52 | 0.50 ± 0.01 | 96.44 | 97.87 ± 1.24 | 1.26 |
|  |  | 5.2 | 5.12 ± 0.03 | 98.52 |  |  |
|  |  | 20.8 | 20.52 ± 0.07 | 98.64 |  |  |
| 0.1 % SDS | | 0.5125 | 0.50 ± 0.01 | 98.05 | 98.27 ± 1.57 | 1.60 |
|  |  | 5.125 | 4.99 ± 0.05 | 97.45 |  |  |
|  |  | 20.5 | 20.36 ± 0.07 | 99.32 |  |  |

**Table S3** Intra- and inter-day precision of CyA determined by HPLC. (*n* = 5)

| C_added_ (μg/mL) | | Intra-day | | Inter-day | |
| --- | --- | --- | --- | --- | --- |
|  |  | **C_detected_ (μg/mL)** | **RSD (%)** | **C_detected_ (μg/mL)** | **RSD (%)** |
| Methanol | 0.99 | 0.98 ± 0.03 | 2.96 | 0.97 ± 0.02 | 2.43 |
|  | 9.9 | 9.48 ± 0.12 | 1.30 | 9.61 ± 0.20 | 2.07 |
|  | 99 | 99.73 ± 1.09 | 1.10 | 99.13 ± 1.46 | 1.48 |
| pH = 1.0 | 0.5025 | 0.53 ± 0.01 | 2.46 | 0.55 ± 0.01 | 2.70 |
|  | 5.025 | 5.03 ± 0.10 | 2.00 | 5.13 ± 0.13 | 2.48 |
|  | 20.1 | 19.70 ± 0.49 | 2.50 | 19.63 ± 0.27 | 1.38 |
| pH = 6.8 | 0.52 | 0.50 ± 0.01 | 2.64 | 0.50 ± 0.01 | 2.83 |
|  | 5.2 | 5.14 ± 0.10 | 1.90 | 5.19 ± 0.13 | 2.46 |
|  | 20.8 | 20.48 ± 0.02 | 0.08 | 20.44 ± 0.10 | 0.48 |
| 0.1 % SDS | 0.5125 | 0.50 ± 0.02 | 2.99 | 0.51 ± 0.01 | 2.10 |
|  | 5.125 | 5.03 ± 0.11 | 2.19 | 4.99 ± 0.12 | 2.49 |
|  | 20.5 | 20.35 ± 0.04 | 0.21 | 20.14 ± 0.13 | 0.67 |

**2.2 Quantification of sorbic acid in two dissolution media by HPLC**

The linear equation was *C* = 0.0078 *A* + 0.1523 (r = 0.9998) within the concentration range of 0.1095 - 219 μg/mL.

- 1. **Quantification of CyA in rat whole blood by HPLC**

CyA and internal standard (CyD) could be well separated from impurities with retention times of 7.3 and 9.8 min, respectively. The *in vivo* linear equation was *C* = 1.194 *As*/*Ai* + 0.2845 (r = 0.9981) where *As* represents the peak area of CyA, *Ai* represents the peak area of CyD within the concentration range of 0.1 - 20 μg/mL. Under the above conditions, the detection limit (*S*/*N* = 3) and the quantitative limit (*S*/*N* = 5) were 0.2 and 0.5 μg/mL, respectively. The accuracy and extraction recovery for samples of low, medium and high concentrations and the precision for within-day and between-day assays met the requirements of *in vitro* determination (shown in **Tables S4, S5** and **Table S6**). Stability of CyA in rat whole blood within 24 h after treatment was good (shown in **Table S7**).

**Table S4** Accuracy of CyA in rat whole blood determined by HPLC. (*n* = 5)

| C_added_ (μg/mL) | C_detected_ (μg/mL) | Recovery (%) | Mean ± SD (%) | RSD (%) |
| --- | --- | --- | --- | --- |
| 0.5 | 0.52 ± 0.02 | 104.03 | 101.09 ± 3.09 | 3.06 |
| 2.0 | 2.00 ± 0.03 | 99.78 |  |  |
| 10.0 | 9.95 ± 0.13 | 99.46 |  |  |

**Table S5** Extraction recovery rate of CyA in rat whole blood determined by HPLC. (*n* = 5)

| C_added_ (μg/mL) | C_detected_ (μg/mL) | Recovery (%) | Mean ± SD (%) | RSD (%) |
| --- | --- | --- | --- | --- |
| 0.5 | 0.32 ± 0.04 | 63.88 | 71.49 ±7.35 | 10.28 |
| 2.0 | 1.51 ± 0.09 | 75.69 |  |  |
| 10.0 | 7.49 ± 0.26 | 74.90 |  |  |

**Table S6** Intra- and inter-day precision of CyA in rat whole blood determined by HPLC. (*n* = 5)

| C_added_ (μg/mL) | Intra-day | | Inter-day | |
| --- | --- | --- | --- | --- |
|  | **C_detected_ (μg/mL)** | **RSD (%)** | **C_detected_ (μg/mL)** | **RSD (%)** |
| 0.5 | 0.52 ± 0.02 | 7.50 | 0.53 ± 0.01 | 5.43 |
| 2.0 | 2.00 ± 0.03 | 1.98 | 1.98 ± 0.06 | 3.53 |
| 10.0 | 9.95 ± 0.13 | 1.40 | 10.00 ± 0.53 | 5.42 |

**Table S7** Solution stability of CyA in rat whole blood within 24 hrs after treatment. (*n* = 6)

| C_added_ (μg/mL) | C_detected_ (μg/mL) | RSD (%) |
| --- | --- | --- |
| 0.5 | 0.51 ± 0.01 | 4.53 |
| 2.0 | 1.99 ± 0.06 | 3.35 |
| 10.0 | 9.99 ± 0.27 | 2.79 |

- 1. **Characterization of different ionic liquids.**
     1. **Characterization of MCILs**


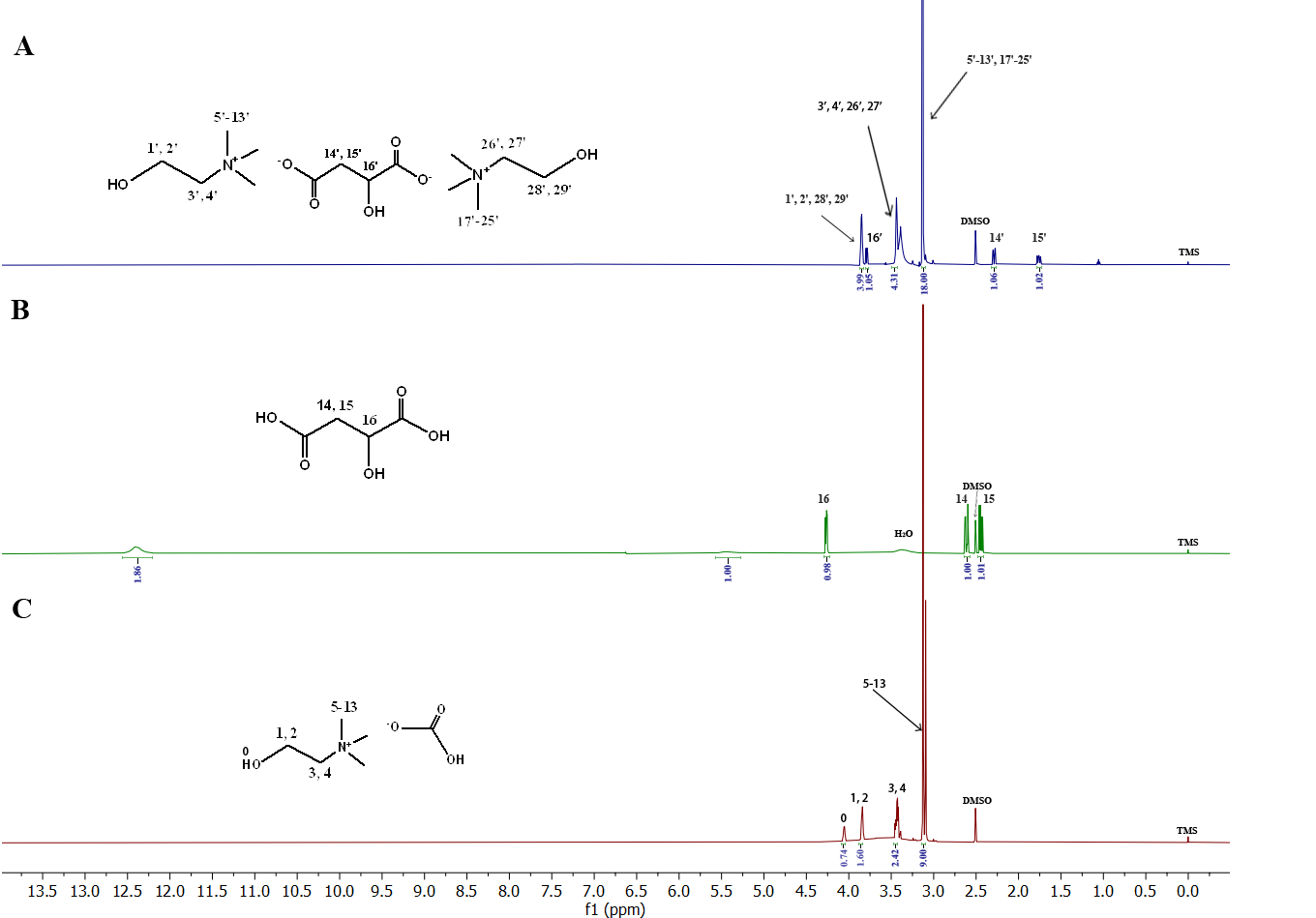


**Figure S1** ^1^H-NMR spectra of MCILs (A), malic acid (B) and choline bicarbonate (C).

- - 1. **Characterization of CCILs**


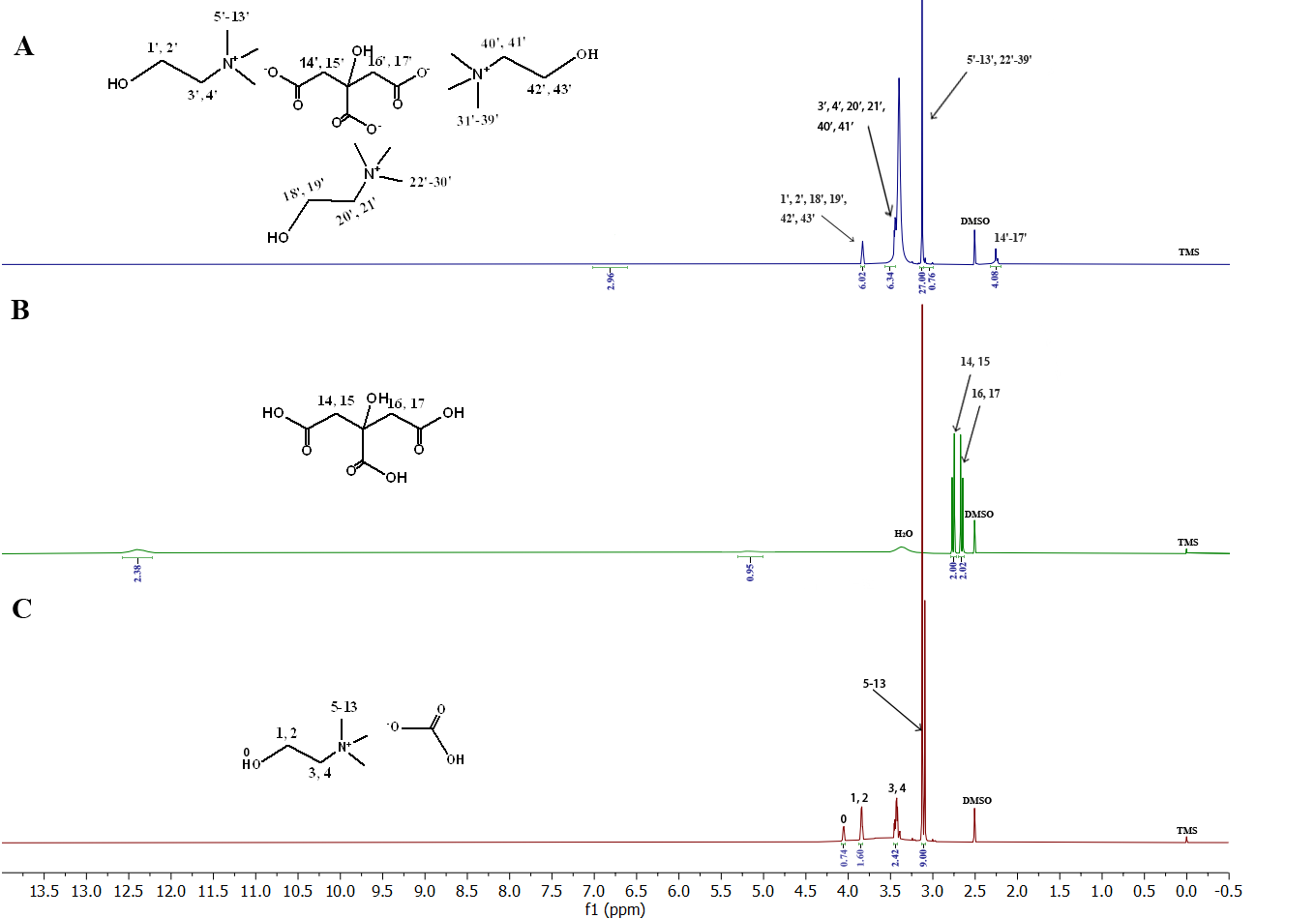


**Figure S2** ^1^H-NMR spectra of CCILs (A), citric acid (B) and choline bicarbonate (C).

- 1. **Solubilization of CyA in different ionic liquids.**

**Table S8** Saturated solubility of CyA in three ionic liquids with different percentages of water.

| Weight ratio (ILs/water) | Saturated solubility of CyA (mg/mL) | | |
| --- | --- | --- | --- |
|  | **MCILs** | **SCILs** | **CCILs** |
| 10/0 | 9.86 | 247.38 | 20.25 |
| 10/1 | 0.36 | 170.69 | 16.42 |
| 10/2 | 0.08 | 148.57 | 2.91 |
| 10/5 | 0.07 | 65.75 | 2.67 |
| 10/10 | 0.05 | 0.38 | 2.24 |
| 10/40 | 0.02 | 0.27 | 1.17 |
| 10/90 | 0.02 | 0.10 | 0.05 |

- 1. **Saturated solubility of CyA in different pH aqueous media.**

**Table S9** Saturated solubility of CyA in different pH aqueous media.

| Media | CyA solubility (μg/mL) |
| --- | --- |
| pH 1.0 | 18.73 |
| pH 2.0 | 17.09 |
| pH 3.0 | 19.80 |
| pH 4.5 | 14.32 |
| pH 6.8 | 13.06 |
| pH 7.0 | 26.44 |
| pH 8.0 | 12.44 |

- 1. **Moisture content**

The determined water content of SCILs was 1.73% ± 0.26%.

- 1. **The stability of enteric CyA@SCIL@MSNs-4**

The CyA content in enteric CyA@SCIL@MSNs-4 were 98.26% ± 1.34% at day 0 and 95.78% ± 0.23% after 10 months storage. The dissolution profiles of CyA from enteric CyA@SCIL@MSNs-4 after 10 months storage (**Figure S3**) were similar to those at day 0, demonstrating the robust stability after long-term storage.


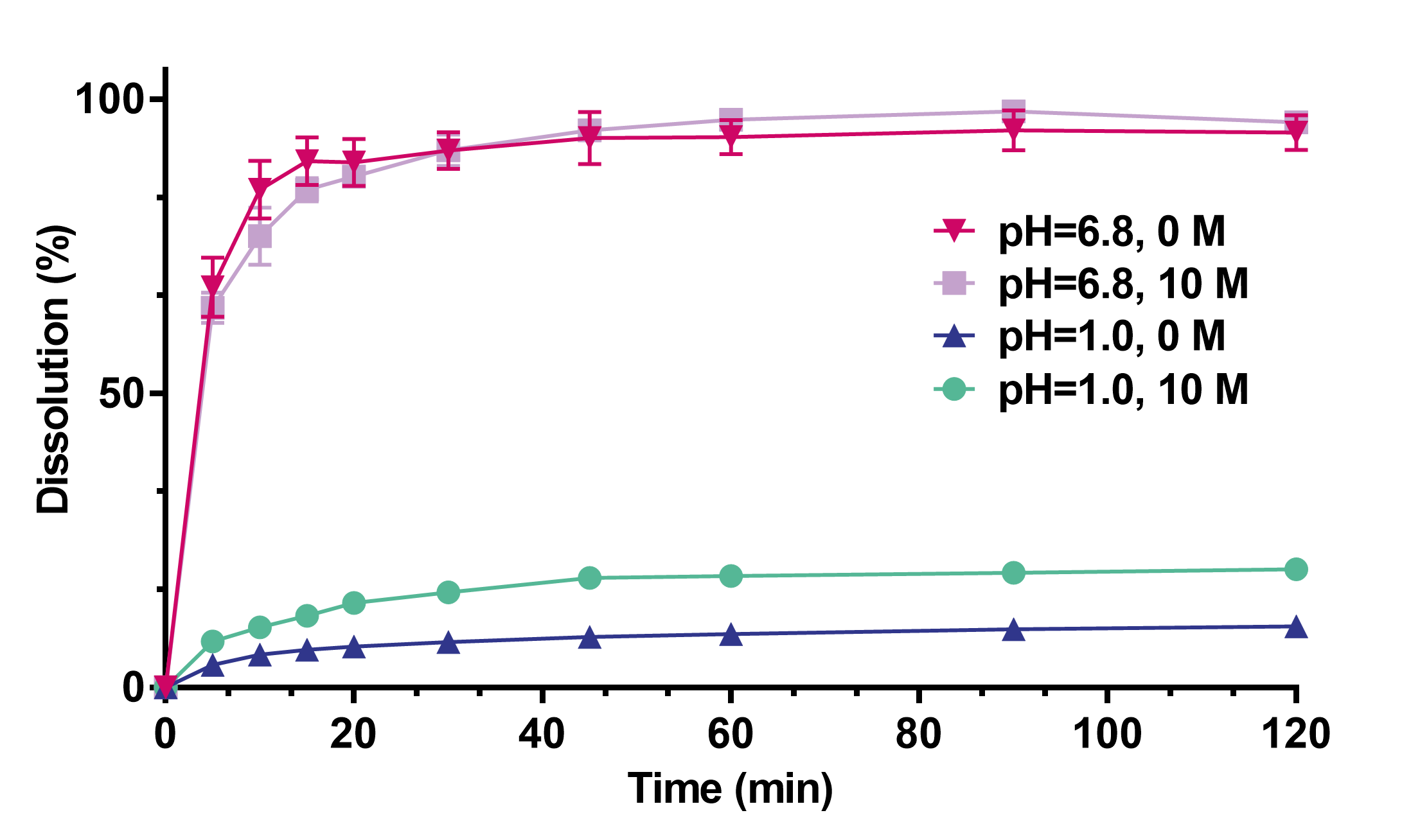


**Figure S3** Dissolution profiles of CyA from enteric CyA@SCIL@MSNs-4 in pH 1.0 hydrochloric acid (containing 0.1% SDS) and pH 6.8 phosphate buffer (containing 0.1% SDS). (*n* = 3)

- 1. **Oral biocompatibility *in vivo***

**Figure S4.** Changes in body weight after 7 days of once-a-day repeat oral dosing. (*n* = 6).

**
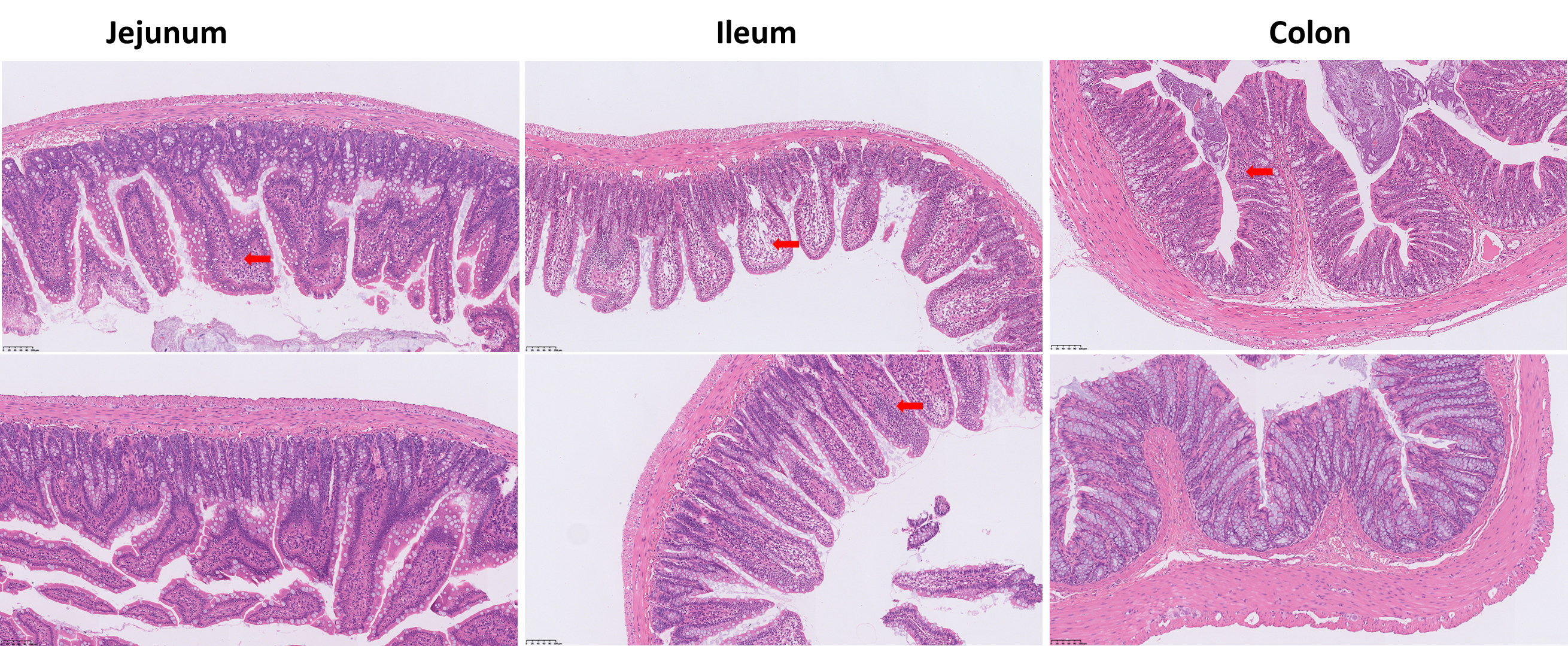
**

**Figure S5.** Representative images of H&E staining for jejunum, ileum and colon sections of Neoral^®^ group (the upper row) and enteric CyA@SCIL@MSNs-4 group (the lower row) (scale bars: 200 μm), *n* = 6 for each group. Red arrows indicated the inflamed sites.

The rats body weight steadily increased in both groups during 7 days of once-a-day repeat oral dosing (**Figure S4**). However, the weight gain of rats in the group of enteric CyA@SCIL@MSNs-4 was significantly more than that of Neoral^®^ group (**Figure S4**), implying enteric CyA@SCIL@MSNs-4 reduced irritation to intestinal tracts compared to Neoral^®^. Physiological symptoms including lethargy, diarrhea, hunched posture, and unkempt fur were not observed in both groups. There existed a few infiltrated sites of immune cells such as neutrophils, lymphocytes, or macrophages in ileum sections among both groups, representing mild tissue inflammation. Also, number of goblet cells decreased slightly in ileum sections among both groups. Nevertheless, the level of irritation to jejunum of enteric CyA@SCIL@MSNs-4 group was slighter than that of Neoral^®^ group. Meanwhile, there were not any tissue inflammation and infiltrated immune cells found in jejunum and colon of enteric CyA@SCIL@MSNs-4-treated animals (**Figure S5**).
